# Multi-mycotoxin analysis using dried blood spots and dried serum spots

**DOI:** 10.1007/s00216-017-0279-9

**Published:** 2017-03-15

**Authors:** Bernd Osteresch, Susana Viegas, Benedikt Cramer, Hans-Ulrich Humpf

**Affiliations:** 1 Institute of Food Chemistry, Westfälische Wilhelms-Universität Münster, Corrensstr. 45, 48149 Münster, Germany; 2 Environment and Health RG, Lisbon School of Health Technology, Polytechnic Institute of Lisbon, Av. D. João II, Lote 4.69.01, Parque das Nações, 1990-096 Lisbon, Portugal; 3 Centro de Investigação em Saúde Pública, Escola Nacional de Saúde Pública, Universidade NOVA de Lisboa, Avenida Padre Cruz, 1600-560 Lisbon, Portugal

**Keywords:** Biomonitoring, Dried blood spot, Dried serum spot, HPLC-MS/MS, Mass spectrometry, Mycotoxin

## Abstract

In this study, a rapid multi-mycotoxin approach was developed for biomonitoring and quantification of 27 important mycotoxins and mycotoxin metabolites in human blood samples. HPLC-MS/MS detection was used for the analysis of dried serum spots (DSS) and dried blood spots (DBS). Detection of aflatoxins (AFB_1_, AFB_2_, AFG_1_, AFG_2_, AFM_1_), trichothecenes (deoxynivalenol, DON; DON-3-glucoronic acid, DON-3-GlcA; T-2; HT-2; and HT-2-4-GlcA), fumonisin B_1_ (FB_1_), ochratoxins (OTA and its thermal degradation product 2’*R*-OTA; OTα; 10-hydroxychratoxin A, 10-OH-OTA), citrinin (CIT and its urinary metabolite dihydrocitrinone, DH-CIT), zearalenone and zearalanone (ZEN, ZAN), altenuene (ALT), alternariols (AOH; alternariol monomethyl ether, AME), enniatins (EnA, EnA_1_, EnB, EnB_1_) and beauvericin (Bea) was validated for two matrices, serum (DSS), and whole blood (DBS). HPLC-MS/MS analysis showed signal suppression as well as signal enhancement due to matrix effects. However, for most analytes LOQs in the lower pg/mL range and excellent recovery rate were achieved using matrix-matched calibration. Besides validation of the method, the analyte stability in DBS and DSS was also investigated. Stability is a main issue for some analytes when the dried samples are stored under common conditions at room temperature. Nevertheless, the developed method was applied to DBS samples of a German cohort (*n* = 50). Besides positive findings of OTA and 2’*R*-OTA, all samples were positive for EnB. This methodical study establishes a validated multi-mycotoxin approach for the detection of 27 mycotoxins and metabolites in dried blood/serum spots based on a fast sample preparation followed by sensitive HPLC-MS/MS analysis.

**Electronic supplementary material:**

The online version of this article (doi:10.1007/s00216-017-0279-9) contains supplementary material, which is available to authorized users.

**Graphical Abstract:**
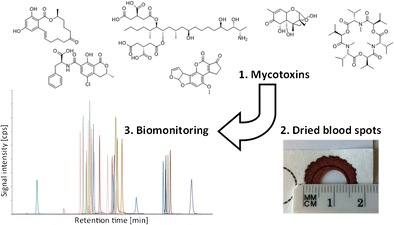
ᅟ

## Introduction

Mycotoxins are toxic secondary metabolites produced by molds contaminating food during production, shipping, processing, or storage [1]. In order to protect consumers’ health maximum levels for food and feed have been set by regulatory authorities [2]. Regulatory attempts are usually based on food contamination and consumption data. However, this approach does not take individual exposure due to personal, regional as well as cultural divergences into account [3]. Biomarker-based approaches are more and more used to assess dietary exposure, as they allow to analyze physiological samples like urine or blood for each test person individually [4]. Various sample preparation and quantification techniques are described for mycotoxins and their metabolites in several physiological samples [5–9]. Main challenges in biomonitoring-based methods are usually low analyte concentrations and matrix interferences during analysis. Recently developed dilute-and-shoot approaches for urine attempt to remove the majority of matrix compounds by chromatographic separation and take advantage of highly sensitive mass spectrometers [10, 11].

For blood analysis, dried blood spots (DBS) currently undergo a comeback concerning medical or forensic issues mainly due to improved detection limits and therefore leading to new fields of application [12, 13]. For example, DBS are suitable for extensive biomonitoring studies of environmental contaminants in humans or animals [14]. Particularly, for animal studies the application of DBS is an effective improvement concerning sample collection as only limited blood volumes are often available for analysis due to the low body weight of small animals [15]. Advantages of DBS compared to conventional blood collection are the minimally invasive sampling, simple sample preparation, easier storage, and shipping as well as the small volume required [16]. Samples can be collected on filter paper cards by heel, ear, or finger pricking as well as by spotting a known blood volume from ampoules after conventional vain puncture [17, 18]. Even if DBS are the standardized basis for medical tests, dried serum spots (DSS) are an additional opportunity for immunological tests and other blood counts. Likewise to DBS, DSS take advantage of simplified storage and shipment conditions [19, 20].

Recently, a DBS method for the detection of ochratoxin A in dried blood spots using HPLC-MS/MS has been published including the optimization of various basic parameters such as spotting volume and hematocrit which did not have a strong influence [21, 22]. Thus, the application of DBS for mycotoxin analysis showed positive findings in all samples for OTA as well as 2’*R*-OTA in the blood of coffee drinkers [21]. Here, we present the further development of this method by the incorporation of 27 relevant mycotoxins and metabolites and its application to serum and blood samples. Compounds monitored were aflatoxins (AFB_1_, AFB_2_, AFG_1_, AFG_2_, AFM_1_), *Alternaria* toxins (ALT, AME, AOH), citrinin (CIT), and its metabolite dihydrocitrinone (DH-CIT). Furthermore, trichothecenes as deoxynivalenol (DON), deoxynivalenol-3-glucuronide (DON-3-GlcA), T-2-toxin (T-2), HT-2-toxin (HT-2), HT-2-toxin-4-glucoronide (HT-2-4-GlcA) have been incorporated. In addition, the structurally related cyclic hexadepsipeptides beauvericin (BEA) and enniatins (EnA, EnA_1_, EnB, EnB_1_) are included. Lastly, fumonisin B_1_ (FB_1_), ochratoxin A, and its thermal degradation product 2’*R*-ochratoxin A as well as ochratoxin α and 10-hydroxyochratoxin A (OTA, 2’*R*-OTA, OTα, 10-HOTA), zearalenone (ZEN), and zearalanone (ZAN) are enclosed [1, 23–25]. Those mycotoxins and metabolites are frequently determined in human fluids such as the urine, human breast milk, and blood, or serum and plasma, respectively [26–29]. Their reliable analysis in a multi-mycotoxin approach for physiological samples is a challenging task but allows to determine the individual mycotoxin exposure of humans and animals.

## Experimental

### Safety consideration

Mycotoxins should be handled with care due to their toxic effects for humans. Blood samples underlie biological hazard in principle. It is mandatory to autoclave all contaminated lab material, e.g., ampoules and paper collection cards, which encountered blood or serum.

### Chemicals and reagents

Acetonitrile (ACN) and acetone were of LC gradient purity (VWR, Darmstadt, Germany). Type 1 laboratory water was used (Resistivity > 18 MΩ, TOC < 10 ppb; Purelab flex, Veolia Water Technologies, Celle, Germany). Formic acid and acetic acid was purchased from Grüssing (Filsum, Germany) with 99.5% purity. Blood collection tubes were MonovetteR® EDTA KE/7.5 mL and Monovette® Serum-Gel from Sarstedt (Nümbrecht, Germany). Whatman 903 protein saver cards™ for sample collection and preparation were acquired from Sigma-Aldrich (Taufkirchen, Germany).

AFB_1/2_, AFG_1/2_, AFM_1_, BEA, CIT, EnA, EnA_1_, EnB, EnB_1_, FB_1_, FB_2_, ZAN, and α-/β-zearalenol were from Sigma-Aldrich. DH-CIT was purchased from AnalytiCon Discovery (Potsdam, Germany). DON, FB_1_, 10-OH-OTA, OTA, OTα, T-2, HT-2, and ZEN were isolated and purified from fungal cultures [30–35]. 2’*R*-OTA was produced by thermal isomerization of OTA [25]. Glucuronic acid conjugates (DON-3-GlcA and HT-2-4-GlcA) were synthesized enzymatically using rat and pig liver microsomes [36]. ALT, AME, and AOH were isolated and purified according to a recently published procedure [37]. Tenuazonic acid was chemically synthesized by Lohrey et al. [38]. Stock solutions were prepared at concentrations of 10 or 20 μg/mL in ACN or ACN/H_2_O and stored at −20 °C in the dark until further use. Purity of in-house produced standards was checked by HPLC-DAD/ELSD (evaporative light scattering detector) and was ≥98%. Structure verification was done via MS and NMR. Aflatoxins B_1/2_ and G_1/2_ were applied as a combined solution at 2 μg/mL for AFB_1_/AFG_1_ and 0.5 μg/mL for AFB_2_/AFG_2_. Two working solutions were prepared at 20-fold concentration of the highest calibration point at each day of measurement in ACN/H_2_O (80:20, *v*/*v*) leading to concentrations of 1 μg/mL for the majority of analytes. For AFB_1_/AFG_1_ the concentrations in the working solution were 200 ng/mL and for AFB_2_/AFG_2_ 50 ng/mL.

### Sample collection

Human blood and serum samples were provided by healthy volunteers giving written consent as participants of biomonitoring studies in Germany (Ethical committee of the University Hospital Münster, Germany, file reference: 2014-187-f-S). Of each sample 100 μL of blood was spotted four times on filter paper cards.

### Sample preparation

Serum was prepared by whole blood collection in Monovette® Serum-Gel tubes and centrifugation at 3000×*g* for 10 min. Afterwards, the supernatant was used as serum sample. For sample preparation, 100 μL of whole blood or serum was pipetted on Whatman 903 protein saver cards™ and dried over night at room temperature to obtain DBS and DSS. In order to assure no overlapping of the DSS, only three of the five marked spotting positions were used when 100 μL serum was applied to the cards, as serum spots show a larger diameter compared do DBS due to lower viscosity. Next, 100 μL DBS or DSS were completely cut out leaving approx. 1 mm space between the cutting edge and the visually detected border of the spot, followed by extraction with 1 mL extraction mixture consisting of water/acetone/acetonitrile (30:35:35, *v*/*v*/*v*) in 2 mL safe-lock tubes for 30 min under sonication. After extraction, an aliquot of 800 μL was transferred into a new 2-mL safe-lock tube and evaporated to dryness at 50 °C under reduced pressure. The residues were reconstituted with water/acetonitrile/acetic acid (95:5:0.1, *v*/*v*/*v*) followed by centrifugation at 22,000×*g* for 10 min and injection of 30 μL of the supernatant into the HPLC-MS/MS system. Sample preparation was performed in duplicate for the volunteer samples and in triplicate for each fortified recovery sample.

### HPLC-MS/MS setup

**Fig. 1:**
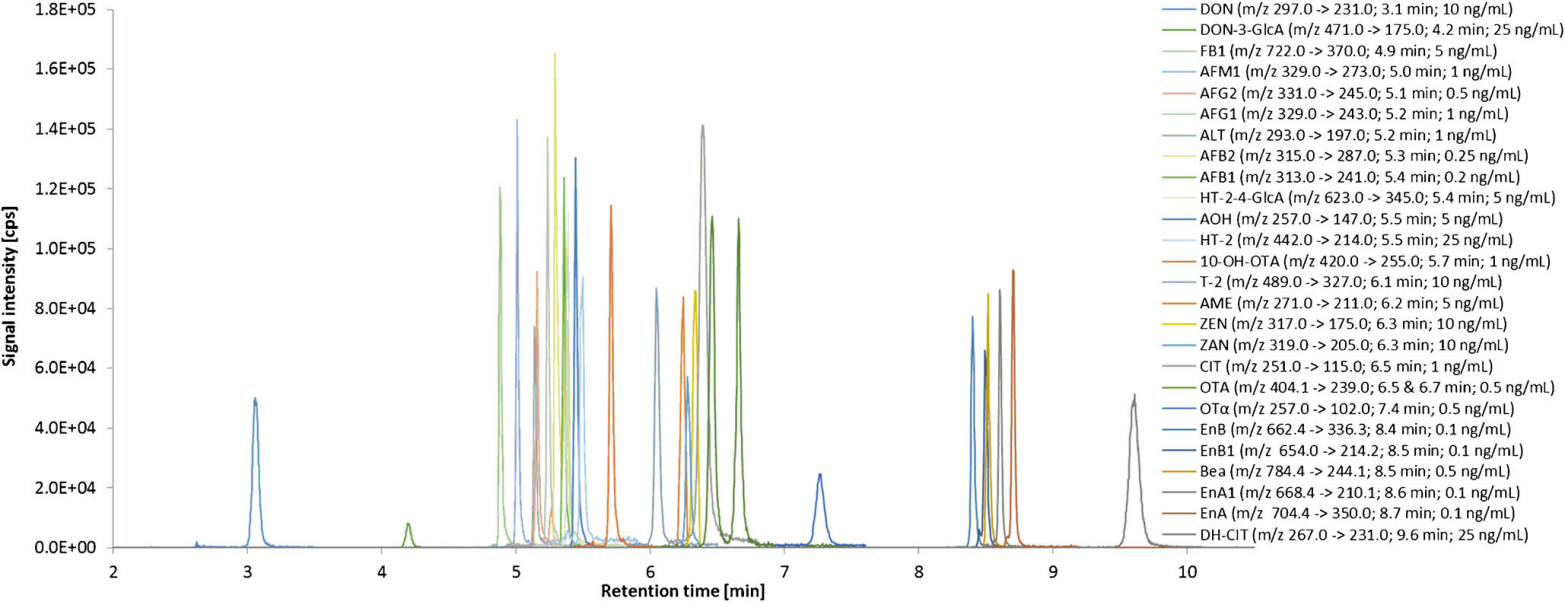
Reconstructed HPLC-MS/MS chromatogram of whole blood (DBS) recovery samples spiked with 27 analytes. Quantifier transition and retention times are given in *brackets* for each signal

Analysis was carried out on a 1260 Infinity LC system (Agilent, Waldbronn, Germany) coupled to a QTRAP 6500 mass spectrometer (SCIEX, Darmstadt, Germany). During method development four different column materials, Nucleodur C_18_ ISIS, Pyramid, Gravity, and Gravity SB (all Macherey-Nagel, Düren, Germany) were compared for retention behavior and peak shape (see Electronic supplementary material). Finally, a Nucleodur C_18_ Gravity SB column (3 μm, 2.0 × 100 mm) equipped with a guard column (2.0 × 4.0 mm) of the same material was used at a column temperature of 45 °C. Eluent A was acetonitrile containing 2% acetic acid and eluent B was water with 0.1% acetic acid. The binary gradient and flow rate was set up as follows: 0 min, 3% A (750 μL/min); 3 min, 15% A (750 μL/min); 4.5 min, 55% A (750 μL/min); 6.0 min, 55% A (850 μL/min); 8.0 min, 100% A (850 μL/min); 10.0 min, 100% A (850 μL/min); 10.1 min, 3% A (750 μL/min); 11.5 min, 3% A (750 μL/min). Electrospray ionization (ESI) was performed in both polarities with +5500 or −4500 V in scheduled multiple reaction monitoring (sMRM™) detection mode. Latter increases the number of data points as less transitions are monitored at the same time. Furthermore, longer dwell times are possible resulting in decreased noise levels and better signal-to-noise ratios. Analyst 1.6.2 software was used for data acquisition. In order to avoid a high entry of unwanted polar compounds into the mass spectrometer, a diverter valve was applied between column and mass spectrometer allowing to discard the first 2.6 min of each chromatographic run before entering the ion source. ESI-source parameters were optimized and set to a source temperature of 500 °C, curtain gas to 40 psi, the collision activated dissociation gas to “high”, GS1 (nebulizer gas) to 45 psi and GS2 (heater gas) to 50 psi. The two MRM transitions which showed the best signal-to-noise ratios were monitored per analyte, the one with the highest signal intensity was selected as quantifier. Table 1 lists detailed HPLC-MS/MS parameters, and Fig. 1 shows a reconstructed HPLC-MS/MS chromatogram with the quantifier transition of each analyte.

**Table 1:** Detailed scheduled multiple reaction monitoring (sMRM) parameters for all mycotoxins quantified

Analyte	Q1 mass [*m*/*z*]^a^	Q3 mass [*m*/*z*]^a^	DP [V]	CE [eV]	Exp. *t*_R_ [min]^b^	Mean *t*_R_ ± SD [min]^c^
AFB_1_	313 [M + H]^+^	241 285	141	51 32	5.40	5.43 ± 0.059
AFB_2_	315 [M + H]^+^	287 115	140	37 91	5.50	5.36 ± 0.043
AFG_1_	329 [M + H]^+^	243 199	35 39	37 66	5.20	5.32 ± 0.058
AFG_2_	331 [M + H]^+^	245 303	52	41 36	5.30	5.21 ± 0.058
AFM_1_	329 [M + H]^+^	273 229	40	34 54	5.10	5.09 ± 0.058
ALT	293 [M + H]^+^	197 152	30	39 47	5.20	5.22 ± 0.056
AME	271 [M − H]^−^	211 213	−44	−45 −50	6.35	6.30 ± 0.106
AOH	257 [M − H]^−^	147 171	−80	−38 −40	5.50	5.50 ± 0.056
Bea	784.4 [M + H]^+^	244.1 262.2	5	37 36	8.60	8.59 ± 0.053
CIT	251 [M + H]^+^	115 91	75	68 54	6.40	6.50 ± 0.107
DH-CIT	267 [M + H]^+^	231 203	54	32 39	9.40	9.62 ± 0.106
DON	297 [M + H]^+^	231 219	70	17 17	3.50	3.18 ± 0.117
DON-3-GlcA	471 [M − H]^−^	175 265	−200	−38 −38	4.50	4.55 ± 0.112
EnA	704.4 [M + Na]^+^	350 231.9	5	68 73	8.80	8.78 ± 0.056
EnA_1_	668.4 [M + H]^+^	210.1 228.3	5	35 35	8.70	8.69 ± 0.057
EnB	640.4 [M + H]^+^ 662.4 [M + Na]^+^	196.2 336.3	50 240	32 63	8.40	8.46 ± 0.058
EnB_1_	654.4 [M + H]^+^	214.2 196.1	5	35 52	8.60	8.56 ± 0.058
FB_1_	722 [M + H]^+^	370 334	125	46 54	4.90	4.91 ± 0.035
10-OH-OTA	420 [M + H]^+^	255 181	65	33 61	5.70	5.78 ± 0.064
HT-2	447 [M + Na]^+^ 442 [M + NH_4_]^+^	345 215	140 50	25 18	5.50	5.55 ± 0.052
HT-2-4-GlcA	623 [M − H]^+^	345 285	100	39 40	5.40	5.44 ± 0.050
OTA/2’*R*-OTA	404.1 [M + H]^+^	239 102	70	31 88	6.70	6.49 ± 0.058^d^ 6.69 ± 0.064
OTα	257 [M + H]^+^	102 193	20	54 37	7.20	7.30 ± 0.110
T-2	489 [M + Na]^+^	327 387	120 145	34 29	6.10	6.15 ± 0.082
ZAN	319 [M − H]^−^	205 161	−156	−31 −37	6.45	6.33 ± 0.104
ZEN	317 [M − H]^−^	175 131	−140	−32 −37	6.45	6.42 ± 0.102

MS transitions are listed for quantifier on top and qualifier below for each analyte. Expected retention time (Exp. *t*_R_) is equivalent to programmed time for sMRM algorithm and set against actual measured mean retention time (mean *t*_R_) followed by standard deviation (SD) of all analyzed calibration and recovery solutions

^a^Quantifier top, qualifier below

^b^Expected retention times, as they were programmed for scheduled MRM algorithm

^c^Analyte mean retention time ± SD [min]

^d^OTA and 2’*R*-OTA show identical fragmentation patterns but are chromatographically separated with OTA eluting before 2’*R*-OTA

### Calibration, recovery, and stability testing

Matrix-matched calibration was applied by adding standards to dried blood spot or dried serum spot extract. As no blood sample without detectable amounts of OTA and EnB was available, a sample with low natural contamination of both compounds was used to evaluate method parameters. OTA validation was performed using the diastereomer 2’*R*-OTA as previously described [21]. The level of EnB in the matrix solution was determined as *c*_EnB, Matrix_ = 0.0361 ± 0.0011 ng/mL via standard addition and taken into account for all further calculations.

Matrix-induced signal suppression and enhancement (SSE) was assessed by calculating the ratio of the slopes of matrix-matched and pure solvent calibration curve according to the following equation (see Tables 2 and 3):

$$ \mathrm{S}\mathrm{S}\mathrm{E}\left[\%\right]=\frac{{\mathrm{slope}}_{\mathrm{matrix}\hbox{-} \mathrm{matched}\;\mathrm{calibration}}}{{\mathrm{slope}}_{\mathrm{pure}\;\mathrm{solvent}\;\mathrm{calibration}}}*100 $$

**Table 2:** Validation parameters for serum matrix (DSS): limit of detection (LOD) is calculated with thresholds of S/N = 3

Analyte	LOD [ng/mL]	LOQ [ng/mL]	Working range [ng/mL]	Regression coefficient (*R* ^2^)	Recovery, low [%] (conc. [ng/mL]) *n* = 3	Recovery, medium [%] (conc. [ng/mL]) *n* = 3	Recovery, high [%] (conc. [ng/mL]) *n* = 3	Average recovery [%] *n* = 9	Interday repeatability [%] *n* = 21	SSE [%]
AFB_1_	0.012	0.05	0.05–2.5	0.9939	106 ± 3.2 (0.1)	101 ± 0.3 (0.2)	99 ± 0.6 (1.0)	102 ± 3.8	10.2	19 ± 5.7
AFB_2_	0.013	0.05	0.05–1.25	0.9965	104 ± 8.4 (0.25)	92 ± 7.1 (0.5)	91 ± 7.2 (1.2)	96 ± 9.5	10.1	125 ± 6.7
AFG_1_	0.021	0.1	0.1–5.0	0.9933	96 ± 3.6 (0.2)	90 ± 7.0 (1.0)	90 ± 4.9 (4.5)	92 ± 6.0	13.5	23 ± 4.7
AFG_2_	0.037	0.125	0.125–1.25	0.9988	90 ± 4.6 (0.25)	89 ± 6.3 (0.5)	90 ± 3.5 (1.2)	89 ± 5.0	7.8	28 ± 7.1
AFM_1_	0.017	0.1	0.1–5.0	0.9999	85 ± 0.7 (0.2)	98 ± 7.3 (1.0)	102 ± 9.3 (4.5)	88 ± 8.5	9.9	29 ± 8.1
ALT	0.147	0.5	0.5–25	0.9999	107 ± 2.2 (1.0)	100 ± 0.6 (5.0)	97 ± 7.3 (10.0)	103 ± 1.6	21.7	48 ± 7.9
AME	0.146	0.5	0.5–25	0.9989	97 ± 7.6 (1.0)	92 ± 5.3 (5.0)	98 ± 4.0 (10.0)	96 ± 6.5	17.9	19 ± 4.1
AOH	0.142	0.5	0.5–25	0.9997	91 ± 2.4 (1.0)	90 ± 3.2 (5.0)	89 ± 2.7 (10.0)	90 ± 2.9	22.7	14 ± 3.7
Bea	0.014	0.05	0.05–5	0.9977	92 ± 6.6 (0.1)	104 ± 6.6 (0.5)	87 ± 9.9 (1.0)	94 ± 5.2	15.0	194 ± 12.6
CIT	0.066	0.25	0.25–25	0.9994	92 ± 6.4 (0.5)	99 ± 0.5 (1.0)	91 ± 7.4 (5.0)	94 ± 6.9	12.5	443 ± 14.4
DH-CIT	0.268	1.0	1.0–50	0.9974	102 ± 3.8 (5.0)	88 ± 7.0 (10.0)	90 ± 2.1 (25.0)	93 ± 7.6	8.9	45 ± 1.7
DON	0.263	1.0	1.0–50	0.9997	103 ± 4.6 (5.0)	59 ± 12.8 (10.0)	70 ± 7.6 (25.0)	77 ± 10.3	19.8	47 ± 5.1
DON-3-GlcA	1.287	5.0	5.0–50	0.9799	129 ± 10.2 (5.0)	139 ± 6.4 (10.0)	120 ± 1.2 (25.0)	130 ± 5.1	20.2	68 ± 6.6
EnA	0.0016	0.01	0.01–1	0.9994	95 ± 10.0 (0.05)	95 ± 6.4 (0.10)	117 ± 7.6 (0.50)	102 ± 13.3	14.9	939 ± 25.6
EnA_1_	0.0055	0.025	0.025–1	0.9989	108 ± 5.8 (0.05)	81 ± 5.4 (0.10)	93 ± 5.0 (0.50)	94 ± 6.1	10.9	302 ± 14.5
EnB	0.0012	0.01	0.01–1	0.9991	129 ± 6.5 (0.05)	114 ± 4.3 (0.10)	99 ± 2.8 (0.50)	114 ± 6.9	21.9	217 ± 13.8
EnB_1_	0.0044	0.025	0.025–1	0.9994	94 ± 1.4 (0.05)	95 ± 2.3 (0.10)	91 ± 8.0 (0.50)	93 ± 5.2	15.8	184 ± 13.8
FB_1_	0.521	2.5	2.5–50	0.9989	61 ± 7.7 (5.0)	64 ± 4.1 (10.0)	58 ± 3.8 (25.0)	61 ± 6.0	9.5	159 ± 12.5
10-OH-OTA	0.015	0.05	0.05–2.5	0.9993	82 ± 5.7 (0.1)	95 ± 2.1 (0.5)	116 ± 1.0 (1.0)	98 ± 14.2	13.5	63 ± 5.6
HT-2	1.344	5.0	5.0–50	0.9986	97 ± 9.9 (5.0)	117 ± 1.9 (10.0)	93 ± 3.9 (25.0)	117 ± 12.2	17.8	35 ± 6.8
HT-2-4-GlcA	0.709	2.5	2.5–50	0.9904	121 ± 4.3 (5.0)	119 ± 7.1 (10.0)	106 ± 3.6 (25.0)	117 ± 8.6	3.9	86 ± 3.8
OTA/2’*R*-OTA	0.012	0.05	0.05–2.5	0.9991	113 ± 2.3 (0.1)	99 ± 0.7 (0.5)	119 ± 2.5 (1.0)	110 ± 8.9	10.0	112 ± 3.4
OTα	0.014	0.05	0.05–2.5	0.9985	92 ± 5.6 (0.1)	105 ± 2.8 (0.5)	108 ± 0.9 (1.0)	102 ± 7.9	9.1	46 ± 10.9
T-2	0.227	0.1	1.0–50	0.9977	108 ± 7.1 (5.0)	118 ± 8.0 (10.0)	110 ± 6.1 (25.0)	112 ± 8.3	7.3	46 ± 6.7
ZAN	0.273	1.0	1.0–50	0.9987	101 ± 1.7 (5.0)	99 ± 2.9 (10.0)	89 ± 2.0 (25.0)	96 ± 5.9	10.6	27 ± 2.1
ZEN	0.294	1.0	1.0–50	0.9995	97 ± 5.6 (5.0)	103 ± 3.7 (10.0)	86 ± 5.5 (25.0)	95 ± 8.8	11.2	17 ± 2.8

For limit of quantification (LOQ), the lowest calibration point was used, which exceeds at least a threshold of S/N = 10. Regression coefficient is shown for each working range, which includes three different recovery samples with low-, medium-, and high-spiking levels. Concentrations of fortified levels are given in brackets for each analyte. Results of recovery performance, interday repeatability, and signal suppression and enhancement (SSE) are reported with their calculated RSD and given in [%]. SSE is calculated by the ratio of the slopes of the matrix-matched and the pure solvent calibration curve

**Table 3:** Validation parameters for whole blood matrix (DBS): limit of detection (LOD) is calculated with thresholds of S/N = 3

Analyte	LOD [ng/mL]	LOQ [ng/mL]	Working range [ng/mL]	Regression coefficient (*R* ^2^)	Recovery, low [%] (conc. [ng/mL]) *n* = 3	Recovery, medium [%] (conc. [ng/mL]) *n* = 3	Recovery, high [%] (conc. [ng/mL]) *n* = 3	Average recovery [%] *n* = 9	Interday repeatability [%] *n* = 21	SSE [%]
AFB_1_	0.0059	0.02	0.02–2.5	0.9928	96 ± 1.1 (0.1)	100 ± 3.6 (0.2)	116 ± 2.8 (1.0)	104 ± 9.1	10.6	51 ± 4.0
AFB_2_	0.0125	0.05	0.05–1.25	0.9980	113 ± 9.9 (0.25)	114 ± 5.0 (0.5)	113 ± 4.5 (1.2)	113 ± 6.9	12.5	423 ± 16.8
AFG_1_	0.0140	0.05	0.05–5.0	0.9968	81 ± 2.8 (0.2)	82 ± 4.2 (1.0)	82 ± 7.4 (4.5)	81 ± 5.6	12.7	49 ± 6.6
AFG_2_	0.0274	0.125	0.125–1.25	0.9989	93 ± 2.8 (0.25)	84 ± 3.9 (0.5)	97 ± 1.2 (1.2)	97 ± 4.3	17.7	54 ± 9.9
AFM_1_	0.0143	0.05	0.05–5.0	0.9909	101 ± 3.5 (0.2)	101 ± 2.8 (1.0)	114 ± 0.9 (4.5)	106 ± 6.7	9.1	57 ± 9.5
ALT	0.081	0.5	0.5–25	0.9995	95 ± 2.3 (1.0)	105 ± 1.9 (5.0)	106 ± 2.6 (10.0)	102 ± 5.5	15.0	39 ± 5.4
AME	0.146	0.5	0.5–25	0.9968	110 ± 1.5 (1.0)	109 ± 6.7 (5.0)	99 ± 2.3 (10.0)	106 ± 6.4	8.4	13 ± 4.6
AOH	0.142	0.5	0.5–25	0.9987	88 ± 4.0 (1.0)	86 ± 1.8 (5.0)	88 ± 2.4 (10.0)	87 ± 3.1	20.4	18 ± 4.4
Bea	0.013	0.05	0.05–5	0.9995	101 ± 9.7 (0.1)	83 ± 4.3 (0.5)	81 ± 3.7 (1.0)	88 ± 5.5	9.4	56 ± 3.4
CIT	0.051	0.25	0.25–25	0.9999	95 ± 8.3 (0.5)	91 ± 4.9 (1.0)	81 ± 2.2 (5.0)	89 ± 8.1	19.3	580 ± 17.7
DH-CIT	0.270	1.0	1.0–50	0.9983	113 ± 1.1 (5.0)	98 ± 3.4 (10.0)	94 ± 0.8 (25.0)	102 ± 8.3	16.5	70 ± 5.2
DON	0.292	1.0	1.0–50	0.9995	114 ± 1.7 (5.0)	78 ± 4.9 (10.0)	80 ± 6.7 (25.0)	90 ± 8.6	21.5	18 ± 4.8
DON-3-GlcA	1.335	5.0	5.0–50	0.9784	194 ± 5.1 (5.0)	242 ± 3.5 (10.0)	311 ± 13.8 (25.0)	249 ± 24.4	18.4	24 ± 8.2
EnA	0.0021	0.01	0.01–1	0.9990	115 ± 2.1 (0.05)	104 ± 7.4 (0.1)	102 ± 9.5 (0.5)	107 ± 8.9	21.6	842 ± 23.2
EnA_1_	0.0029	0.01	0.01–1	0.9988	103 ± 3.1 (0.05)	86 ± 9.0 (0.1)	82 ± 6.0 (0.5)	90 ± 5.6	12.2	471 ± 37.1
EnB	0.0013	0.005	0.005–1	0.9998	116 ± 2.8 (0.05)	114 ± 5.3 (0.1)	89 ± 7.0 (0.5)	106 ± 6.7	16.1	259 ± 14.0
EnB_1_	0.0036	0.025	0.025–1	0.9991	103 ± 4.0 (0.05)	94 ± 5.8 (0.1)	82 ± 2.4 (0.5)	93 ± 9.5	11.8	275 ± 14.0
FB_1_	0.627	2.5	2.5–50	0.9962	87 ± 0.5 (5.0)	99 ± 4.0 (10.0)	106 ± 2.3 (25.0)	97 ± 8.5	18.3	298 ± 14.7
10-OH-OTA	0.013	0.05	0.05–2.5	0.9998	104 ± 2.8 (0.1)	98 ± 0.6 (0.5)	117 ± 1.5 (1.0)	110 ± 8.1	8.9	74 ± 5.4
HT-2	1.396	5.0	5.0–50	0.9979	86 ± 8.4 (5.0)	92 ± 8.9 (10.0)	84 ± 2.8 (25.0)	87 ± 8.1	9.3	23 ± 6.2
HT-2-4-GlcA	0.713	2.5	2.5–50	0.9903	134 ± 3.9 (5.0)	130 ± 7.2 (10.0)	133 ± 6.4 (25.0)	132 ± 6.2	14.8	68 ± 8.3
OTA/2’*R*-OTA	0.014	0.05	0.05–2.5	0.9992	100 ± 1.4 (0.1)	101 ± 1.6 (0.5)	108 ± 5.4 (1.0)	103 ± 4.9	9.8	111 ± 8.1
OTα	0.014	0.05	0.05–2.5	0.9999	94 ± 3.6 (0.1)	89 ± 5.4 (0.5)	101 ± 1.2 (1.0)	95 ± 6.0	12.7	61 ± 9.7
T-2	0.205	1.0	1.0–50	0.9988	103 ± 7.8 (5.0)	114 ± 4.6 (10.0)	128 ± 7.1 (25.0)	115 ± 12.0	13.3	35 ± 6.9
ZAN	0.277	1.0	1.0–50	0.9985	100 ± 3.6 (5.0)	103 ± 1.7 (10.0)	113 ± 4.7 (25.0)	105 ± 6.5	17.2	23 ± 2.7
ZEN	0.289	1.0	1.0–50	0.9947	101 ± 3.1 (5.0)	103 ± 3.4 (10.0)	114 ± 8.9 (25.0)	106 ± 8.3	10.2	14 ± 2.2

For limit of quantification (LOQ), the lowest calibration point was used, which exceeds at least a threshold of S/N = 10. Regression coefficient is shown for each working range, which includes three different recovery samples with low-, medium-, and high-spiking levels. Concentrations of fortified levels are given in brackets for each analyte. Results of recovery performance, interday repeatability, and signal suppression and enhancement (SSE) are reported with their calculated RSD and given in [%]. SSE is calculated by the ratio of the slopes of the matrix-matched and the pure solvent calibration curve

Values of 100% correspond to no impairment of analyte detection by matrix while SSE > 100% describes a signal enhancement due to a positive matrix effect and vice versa.

For every analyte, a calibration curve containing at least six points in the working range (Tables 2 and 3) was generated by dilution of the working solutions. Calibration solutions were freshly prepared for every measurement day. Reproducibility was determined by analyzing one real sample of the previously mentioned German sample cohort, naturally containing OTA, 2’*R*-OTA, and EnB, as quality control sample. The determination of LOD and LOQ was based on a signal to noise ratio (S/N) of 3 for LOD and S/N of 10 for LOQ. The calculated LODs are given in Tables 2 and 3.

Recovery rates were determined by spotting blood and serum samples spiked with low, medium, and high analyte concentrations of the working range on DBS cards, followed by standard sample preparation procedure as described above. Three independent replicates for each concentration were prepared, analyzed, and quantified via the respective matrix-matched calibration for calculation of the recovery. Interday performance was evaluated by analysis of fortified recovery samples in both matrices on three different days. On the first day, the samples were worked up in triplicate and on the other 2 days in duplicate for all spiking levels. Detailed data of spiked concentrations, single and averaged results, and relative standard deviations are shown in Tables 2 and 3.

Stability testing of DBS/DSS was done with fortified samples which were stored at room temperature (*T* = 20 °C) and in the dark, as these parameters are used for storage and shipping of DBS when pharmaceutical and medical analysis are applied. The samples were analyzed in duplicate for low, medium, and high spiking levels after storage for 1-10 weeks. Based on these values, mean relative degradation rates were calculated by comparison to freshly prepared recovery samples. Furthermore, two sets of spiked samples were prepared up to 24 weeks and stored at 4 °C in a fridge as well as in a freezer at −18 °C. The batches were kept in plastic boxes until analysis. Humidity during storage in laboratory and fridge was monitored by a hygro-/thermometer and was consistent at 60 ± 10%.

## Results and discussion

In order to develop a multi-mycotoxin method based on dried blood spots (DBS) and dried serum spots (DSS), HPLC-MS/MS analysis of 27 mycotoxins and metabolites was performed in positive and negative mode with electrospray ionization and scheduled multiple reaction monitoring (sMRM™). Analytes have been selected based on their occurrence, toxicological relevance, and availability [1, 23–29]. Polarity of the compounds was not considered as an important criterion for this study despite the fact that glucuronides are usually more relevant biomarkers for urinary exposure assessment than for blood-based human biomonitoring. However, kinetic studies in humans or animals might require the detection of these polar metabolites in order to investigate the metabolic activity and biotransformation rates of individuals.

### Sample preparation

For sampling, the previously developed method [21] was extended and optimized to cover a larger set of 27 analytes and to include serum samples. A defined blood or serum volume of 100 μL was spotted on standard filter paper cards, followed by drying, cutting the entire spot out of the paper, and an extraction with a mixture containing 35% acetonitrile, 35% acetone, and 30% water. After the extraction, an aliquot of the extract was evaporated to dryness, reconstituted, and centrifuged, leading to a clear and colorless solution. During optimization of the extraction procedure, it was observed that the applied centrifugal force has a strong impact on the subsequent HPLC-MS/MS analysis. Centrifugation at 22,000×*g* was superior in matrix removal compared to the previously applied 3000×*g*. For example, centrifugation at 22,000×*g* yielded a higher signal intensity and a lower S/N ratio for FB_1_ (see Fig. S1, Electronic Supplementary Material (ESM)).

### Optimization of the HPLC-MS/MS conditions

Special attention was paid to column selection, solvent additive and gradient to optimize peak shape, and signal response for all compounds from this heterogeneous group of analytes. Besides Nucleodur C_18_ Gravity SB, columns of the same sizes packed with ISIS, Pyramid and Gravity materials were evaluated. Matrix compounds in the extracted blood spot solutions strongly influence analyte separation while simultaneously interfering mass spectrometric detection by falsely triggering the measured transitions. Therefore, reducing the noise level closely to the expected retention times of the substances was mandatory. Another point was the baseline separation of OTA and 2’*R*-OTA within a short LC gradient as both isomers show the same MRM transitions. Both aims were preferable achieved by use of Gravity SB material when extracted matrix solutions spiked with the analytes were injected. The selected column material showed good retention properties for all analytes, in particular for OTα and DH-CIT which usually elute near the parent compounds OTA and CIT, respectively. Additionally, previously used formic acid as eluent additive was replaced by acetic acid. Despite being the weaker acid, chromatographic separation was still excellent. The pH gradient of pH 3.5 to pH 2 caused by fortified concentrations of 0.1% acetic acid in water and 2% acetic acid in acetonitrile, respectively, led to improved peak shapes for DON as well as DH-CIT. Moreover, the weaker protonation properties resulted in higher ionization yields and therefore increased LODs. For instance, the chromatograms in Fig. S2 (ESM) demonstrate the influence of eluent additive on signal intensity and S/N ratio.

Figure 1 shows a reconstructed HPLC-MS/MS chromatogram of spiked DBS recovery samples including all 27 compounds. All analytes and their phase-II-metabolites as well as OTA and 2’*R*-OTA were baseline separated to ensure no detection of artifacts. Chromatographic retention of the first eluting mycotoxin DON was adjusted to about 3.1 min in order to discard unwanted compounds by a diverter valve. Furthermore, to achieve maximum sensitivity with short dwell times, scheduled MRM was applied, reducing the detection window for every MRM to 30 s around the expected retention time.

Table 1 lists all detailed parameters for MS/MS detection. Moreover, programmed expected retention times are shown and set against those actually determined of all analyzed calibration and recovery standard solutions. During this analysis, over a period of 12 weeks, retention time shifts below ±7 s were observed, providing the possibility to apply scheduled MRM to achieve maximum sensitivity with short dwell times. The windows for every MRM were set at 30 s, except for the first and last eluting compounds DON and DH-CIT as it was expected that both would be the least accurately eluting. In fact, these analytes had some of the highest standard deviations concerning mean retention times as demonstrated in Table 1. However, with a variation of about ±6 s for DH-CIT, a retention time window of 30 s would have also been sufficiently broad.

Since matrix effects are a challenge in mass spectrometric detection, the aim was to obtain a matrix effect at a maximum of 10% of its intensity compared to neat eluent solutions in order to decrease the limit of detection as low as possible. Thus, an injection volume of 30 μL compromises on absolute signal intensity as well as matrix suppression (Tables 2 and 3).

### Blood samples for matrix-matched calibration and quality control

**Fig. 2:**
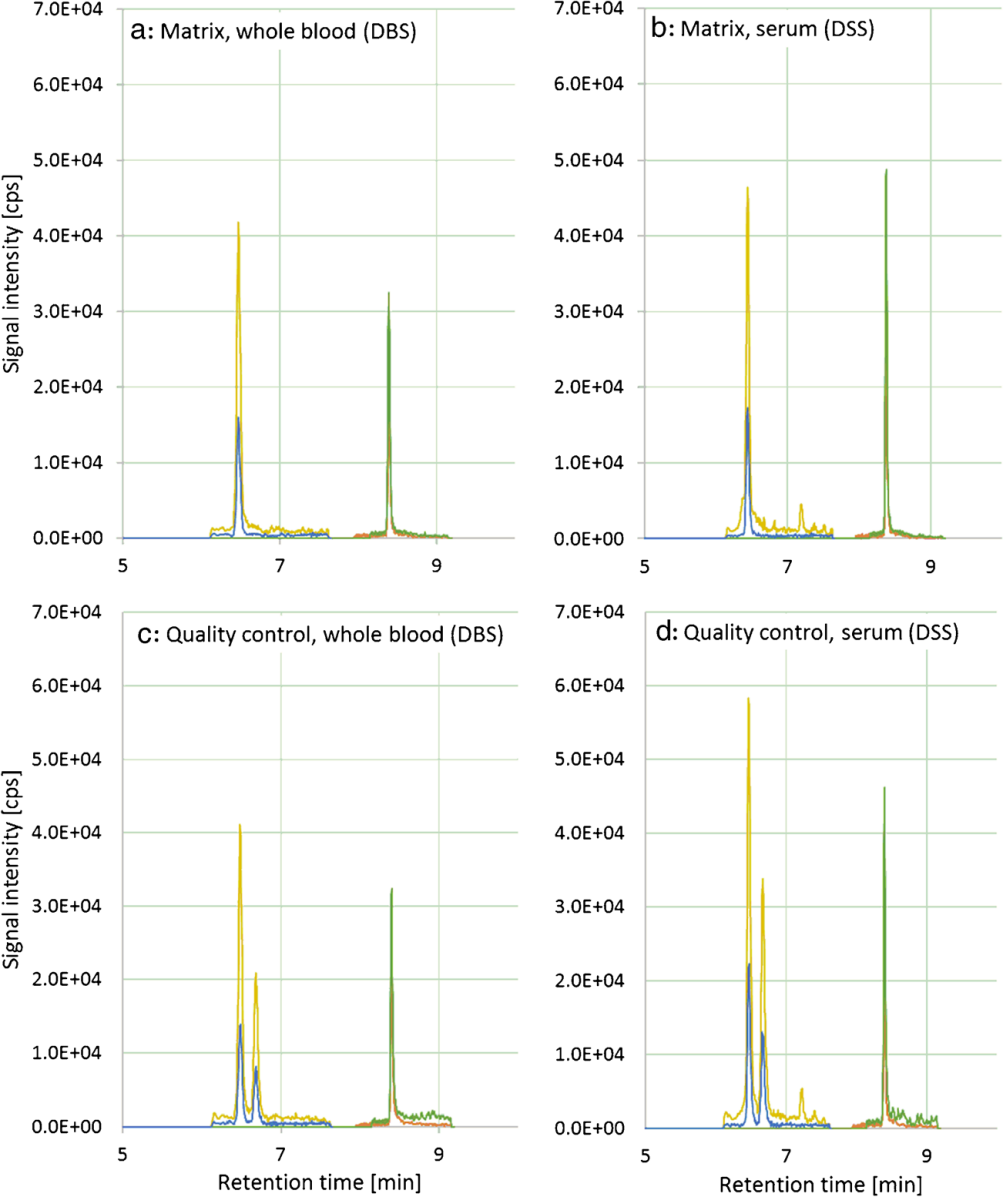
Extracted ion chromatograms of OTA/2’*R*-OTA (*yellow transition m*/*z* 404.1 → 239.0, *blue transition m*/*z* 404.1 → 102.0) and EnB (*green transition m*/*z* 640.4 → 196.2, *orange transition m*/*z* 662.4 → 336.3) in whole blood (**a** and **c**) and serum (**b** and **d**). **a**, **b** Analyte signals for the used matrix solutions containing 0.291 ng/mL OTA and 0.0349 ng/mL EnB. **c**, **d** A real sample which was used as quality control sample containing 0.283 ng/mL OTA, 0.193 ng/mL 2’*R* OTA, and 0.0366 ng/mL EnB

As no blank matrix solution was available, a blood sample with a low natural concentration of OTA and EnB was used for matrix-matched calibration. Figure 2 shows in sections A and B the extracted ion traces for both analytes in whole blood and serum matrix. The used matrix solution lacked the presence of 2’*R*-OTA, which was used for the determination of validation parameters of OTA. Chromatograms C and D in Fig. 2 show the ion traces of a human blood sample containing OTA, 2’*R*-OTA, and EnB which was used as quality control sample.

The diastereomers OTA and 2’*R*-OTA are baseline separated and show the same ratio of the signal transition intensities in both processed whole blood and serum samples matrices. Reproducibility was investigated by the analysis of control samples over a period of 7 days for whole blood and serum. For this, a sample naturally contaminated with OTA, 2’*R*-OTA, and EnB was chosen for performance control in order to provide results most closely to available samples. Therefore, quality control samples were freshly prepared and analyzed on each measurement day and reproducibility was accepted with 7.5% for OTA, 7.2% for 2’*R-*OTA, and 4.2% for EnB, consequently.

### Validation results

Validation experiments were carried out by analyzing linearity, matrix effects, recovery, limit of detection (LOD), and limit of quantification (LOQ) for the serum (Table 2) and whole blood (Table 3) as matrices. Determination of LOD and LOQ was made via linear regression of mean signal to noise ratios of the four lowest matrix calibration standards of each compound. Matrix effect (SSE) calculation was done by comparison of signal intensities in aqueous and matrix solutions by means of the slopes of both calibration curves (see Experimental section for details). Furthermore, the chromatograms indicate no distinct difference between the two matrices concerning the noise levels around the signals (Fig. 2).

Best limits of detection were achieved for the enniatins at about 0.001-0.006 ng/mL in both matrices. Next, the aflatoxins, beauvericin, CIT, 10-OH-OTA, OTα, and OTA/2’*R*-OTA showed good LODs with calculated values up to 0.015 ng/mL. Poorest responses have been determined for HT-2 and DON-3-GlcA with about 1.3–1.4 ng/mL for both analytes. Regression coefficients (*R* ^2^) of 0.9999 to 0.9784 were accomplished by linear regression analysis. Maximum calibration concentrations were set at 50 ng/mL as higher blood contaminations were not anticipated. The LOQ values obtained with this new method indicate that the DBS-based sample preparation technique in combination with modern mass spectrometers is able to reach LOQ values comparable to those of previously published analyte-specific methods. For example, current OTA detection methods based on liquid-liquid extraction of the plasma, urine, or human breast milk lead to similar LOQs of 0.03 ng/mL [39]. Moreover, quantification of CIT can be carried out by immunoaffinity column cleanup resulting in a LOQ of 0.15 ng/mL for blood plasma samples [7]. Analysis of beauvericin and enniatins in pig plasma using HPLC-MS/MS after solid phase extraction reached LOQs of 0.1–0.2 ng/mL which are about one decade higher than the values achieved here (Table 3). Similar method parameters were acquired when human plasma samples were analyzed [5].

Recovery rates were determined by spiking blood and serum samples with low, medium, and high analyte concentrations (see Tables 2 and 3 for spiking levels). As shown in Tables 2 and 3, average recovery rates of 80 to 120% were achieved for 24 out of 27 mycotoxins in the serum and whole blood. FB_1_ and DON reached lower recovery rates of 61 ± 6.0 and 77 ± 10.3%, whereas DON-3-GlcA showed a slightly higher recovery rate of 130 ± 5.1% in serum matrix. In whole blood, the lowest recovery rate was obtained for AFG_1_ with 81 ± 5.6% and the highest with 132 ± 6.2% for HT-2-4-GlcA.

For DON-3-GlcA, the determined mean recovery in DBS was 249%, which is out of the range for reliable quantitative analysis. The high polarity of this compound as well as the low slope of the linear calibration curve due to strong matrix effects are possible explanations. Carry-over effects through the injection system could be excluded as injections of blank solutions after the highest standards were always negative for all compounds. Besides DON-3-GLcA, also DON-15-GlcA is reported as main metabolite of DON in humans [28, 40–43]. Both compounds coelute on most chromatographic systems and show mostly identical fragmentation behavior. As only DON-3-GlcA was available, this was used as reference and an accidental detection of coeluting DON-15-GlcA was accepted.

Matrix effects due to signal suppression or enhancement are a major concern and a well-known phenomenon in mass spectrometry. Consequently, its consideration is recommended for the establishment of new analytical methods. For example, matrix suppression down to 40% signal intensity for CIT can be determined in urine samples even after liquid-liquid extraction followed by solid phase extraction [44]. Therefore, high signal suppression due to only little matrix removal was expected and observed for most of the analytes in both matrices, as it is often described for multi-analyte biomonitoring methods [11, 45].

Among all compounds AOH undergoes the strongest matrix effect in whole blood resulting in a signal intensity of only 14% compared to the standard solution in neat solvent. The lowest signal intensity in the serum was observed for alternariol monomethyl ether which was only 13% compared to the standard solution. OTA and 2’*R*-OTA show in both matrices only minor alteration concerning signal intensity compared to blank solutions. In contrast, FB_1_, CIT, and the enniatins undergo strong signal enhancement, which has particularly for EnB already been reported [46]. Enniatins stand out with positive matrix effects of up to 939% for EnA in processed serum matrix. Interestingly, beauvericin shows a decent signal suppression when detected in whole blood and an increased signal intensity in serum matrix (SSE of 56 and 194%, respectively).

Although the levels of SSEs are severe for some analytes, the matrix effects are highly reproducible as can be seen by their relative standard deviations (RSD). For example, calibration curves for EnA and AME are shown in Fig. S3 (ESM). For the calculations of RSD, five sets of matrix-matched calibration and neat solvent calibration curves were used (Tables 2 and 3). As a result, only slight differences between both matrices were observed for most analytes. Endogenous blood compounds are assumed to be co-extracted and to interfere mass spectrometric detection. Due to the lack of blood cells, we expected a lower matrix influence of serum compared to whole blood; however, the results do not support this. Erythrocytes appear to be intact but are distorted from dehydration in DBS samples [47]. However, it can be supposed that cells are destroyed during the extraction procedure by applying organic solvents as well as sonication. In fact, not all analytes show comparable SSE in both extracted matrices besides beauvericin. Nevertheless, determined recovery rates are comparable for both matrices. In order to study any influence of different matrix samples on DBS analysis, five randomly chosen blood matrices from the study cohort (*n* = 50) were fortified with the highest spiking level in triplicate. The results are shown in ESM Table S1 and no significant differences regarding the average recovery rate compared to the prior obtained results were observed (*P* > 0.05). In summary, the developed method achieves reliable validation parameters for mycotoxin detection in DSS and DBS including a broad range of analyte polarities. The developed method accomplishes a stable and robust quantification performance by use of matrix-matched calibration without the need of isotopically labelled standards, which are not commercially available for most of the mycotoxin metabolites as internal standards.

### Stability testing

A crucial point in DBS and DSS analysis is the stability of the analytes on the filter cards. Especially as DBS cards are usually handled and shipped at room temperature, a certain ageing of the sample material can be expected. Thus, the next step in method validation was stability testing of the analytes in DBS and DSS matrices. On the basis of fortified recovery samples, relative recovery rates were investigated by storing the DSS and DBS cards in the dark at room temperature (*T* = 20 °C), at 4 °C as well as −18 °C.

The stability of all 27 compounds was analyzed and the results after storage for 1, 5, and 10 weeks at room temperature and after storage for 24 weeks at 4 and −18 °C are summarized in Table 4 for whole blood (the results for serum can be found in Table S2 (ESM) as no clear difference between both DBS and DSS matrix was observed when samples were stored at 4 or −18 °C). Detailed relative recovery rates for storage at 20 °C for 1–10 weeks are shown in Figs. S4 and S5 (ESM). Besides OTA/2’*R*-OTA, all other compounds showed a time-dependent degradation at room temperature. Nearly all mean recovery rates decrease already within the first week after sample collection (Table 4). The enniatins, HT-2/HT-2-4-GlcA as well as 10-OH-OTA and OTα show a moderate reduction over the 10-week period resulting in remaining toxin concentrations of 37–75% after 10 weeks. The other compounds undergo a relatively fast degradation under these typical storage conditions to only 4–37% of the original concentration. This trend can be observed for both matrices, DBS as well as DSS (for the DSS results see Table S2, ESM). However, storage for shorter periods like 1 week at room temperature, a time frame sufficient for typical sample shipping, still provides recoveries of 61% for DH-CIT, 63% for AFG_1,_ and for all other compounds at least 74% in serum. In whole blood, after 1 week only DH-CIT (45%), AOH (64%), and ALT (65%) show higher degradation, while all other mycotoxins or metabolites show at least a recovery of 75% of the original concentration.

**Table 4:**
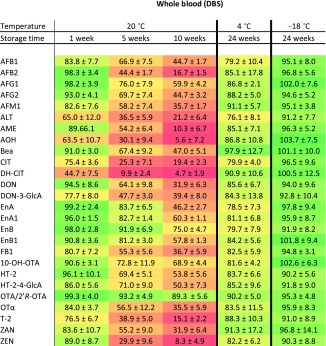
Average relative analyte concentration ± RSD [%] of mycotoxins when stored as dried blood spots for 1, 5, and 10 weeks at room temperature and 24 weeks at 4 and −18 °C in the dark

Freshly prepared recovery solutions were set at 100% and corresponding recovery rate after storage are depicted. Color gradient emphasizes the level of nearly no degradation (≥95%, green) towards the highest (≤ 50%, red) with color steps of 5%

**Fig. 3:**
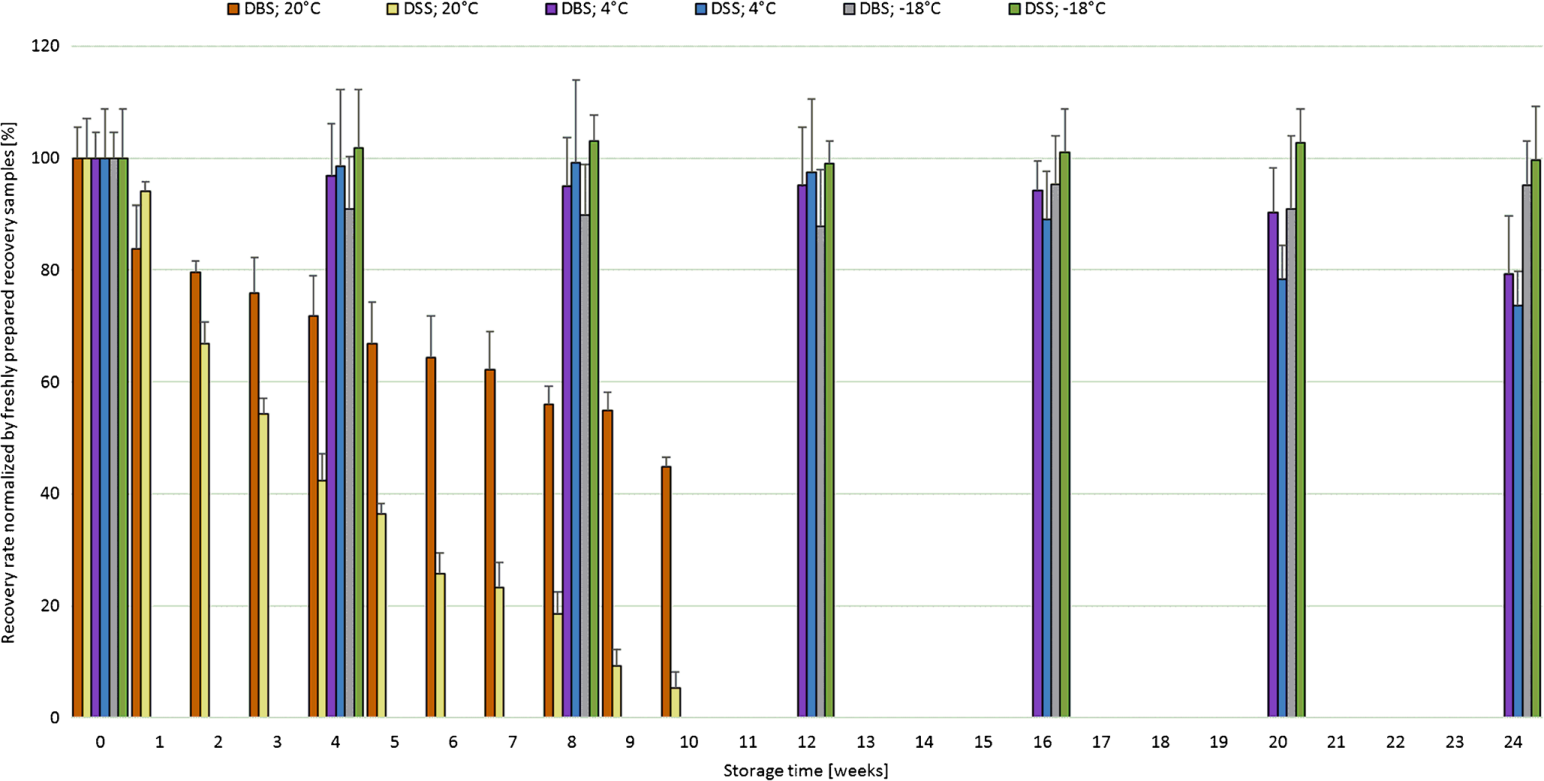
Relative recovery rates of AFB_1_ in fortified DBS and DSS; storage at room temperature (20 °C) for 1-10 weeks and at 4 °C as well as −18 °C for 4-24 weeks

Although room temperature is the typical condition for shipping and storage of DBS in clinical studies, the relative recovery rates of fortified DBS and DSS were investigated when the samples were stored at 4 and −18 °C up to 24 weeks. As representative example, stability curves for AFB_1_ under all storage conditions are shown in Fig. 3. The summarized results in Table 4 clearly demonstrate that most analytes are relatively stable when stored at 4 or −18 °C. After 24 weeks of storage, all recovery rates were still above 76 and 88% at 4 and −18 °C, respectively. During all studies, humidity was held at a constant level of 60 ± 10%, which corresponds to typical laboratory conditions.

Thus, we recommend to store the dried samples under light exclusion in sealed containers at −18 °C to minimize potential degradation of the mycotoxins as discussed above. To our knowledge, this is the first evaluation of mycotoxin stability in dried physiological samples such as DBS and DSS. The results clearly show that sample storage under optimal conditions is safe but can become an issue when temperature above 4 °C is reached.

### Biomonitoring of blood samples

The developed multi-mycotoxin method was applied for the analysis of blood samples of a German sample cohort which have already been analyzed in our first DBS-study for OTA and 2’*R*-OTA [21]. For multi-mycotoxin analysis of this sample cohort, DBS (*n* = 50) were prepared in duplicate and quantified according to the matrix-matched calibration parameters. Besides OTA and EnB, no other mycotoxins or metabolites were detectable. Although DON and especially its phase-II-metabolite are often detectable in human urine samples from Germany [11], it seems that DON is rapidly converted and excreted as it is not detectable in DBS samples. Figure 2 shows typical HPLC-MS/MS chromatograms as the quality control is one sample out of the German sample cohort.

Enniatin B was detectable in all samples with a mean value of 0.0367 ± 0.0179 ng/mL and a median of 0.0330 ng/mL. The lowest and highest concentrations were with 0.0144–0.1071 ng/mL in the calibration range. Intake of EnB by food is supposed to be the main reason of exposure. However, risk assessment is currently not possible due to the lack of data for dietary exposure as well as toxicity, so that no TDI is established yet [48]. Considering the low concentrations of EnB in the analyzed DBS, this study contributes results for the presence of EnB in human blood samples as it has already been reported in urine and breast milk samples [11, 25]. Previously quantified OTA and 2’*R*-OTA [21] were reanalyzed by this multi-mycotoxin method. OTA was found in all samples as well as 2’*R*-OTA in the same samples as before. However, some samples (*n* = 9 out of 34) showed signals for 2’*R*-OTA between LOD and LOQ due to slightly lower sensitivity of this multi-mycotoxin method. Therefore, all positive findings were confirmed. Figure S6 (ESM) demonstrates the correlation between the analyte amounts calculated by both methods. The arithmetic mean of OTA (*n* = 50) by [21] with *c*_OTA_ = 0.211 ± 0.064 ng/mL is matching to *c*_OTA_ = 0.207 ± 0.063 ng/mL. Likewise, the average values for 2’*R*-OTA are *c*_2’*R*-OTA_ = 0.112 ± 0.092 ng/mL (*n* = 34) and *c*_2’*R*-OTA_ = 0.143 ± 0.100 ng/mL (*n* = 25), respectively. Latter value is certainly higher because the amounts of nine samples which show signals > LOD are not considered. Calculated regression coefficients of *R* ^2^ = 0.9043 (OTA) and *R* ^2^ = 0.9512 (2’*R*-OTA) support the comparability of both sample preparations (Fig. S6, ESM). Thus, the results clearly demonstrate the comparability of both methods for the detection and quantification of OTA and 2’*R*-OTA in DBS.

## Conclusion

A DBS- and DSS-based sample preparation technique for the detection of various mycotoxins and metabolites was established and validated. The developed multi-mycotoxin method allows the simultaneous detection and quantification of 27 analytes by HPLC-MS/MS in dried whole blood (DBS) and serum spots (DSS). On the basis of previous DBS studies [21, 22], the improved method has the same advantages such as simplified storage and shipment conditions as well as reduced use of chemicals and materials compared to other sampling techniques.

The developed DSS and DBS-based method was evaluated for linearity, limit of detection, and quantification, robustness, recovery, and stability of fortified samples. It could be shown that DSS and DBS are a suitable sampling technique for the detection of multi-mycotoxin exposure due to adequate validation parameters for most of the incorporated mycotoxins and metabolites. Complications arise from stability issues which have to be taken into account and for this reason storage of samples at −18 °C is recommended. The developed method was applied to DBS samples of a German cohort, showing that besides OTA [21], all samples (*n* = 50) were positive for EnB with mean levels of 0.0367 ng/mL. All in all, a novel application for the use of DSS and DBS for multi-mycotoxin exposure studies was developed and validated, proofing the effective performance characteristics needed for biomarker or biomonitoring approaches. As some mycotoxins or metabolites are mainly detectable in urine (e.g., DON-3-GlcA) [10, 11] and others such as OTA, 2’*R*-OTA, EnB mainly in the blood (Fig. 2), the analysis of both matrices is recommended to evaluate the human and animal exposure to mycotoxins. Besides dilute-and-shoot approaches used for urine samples, the use of DBS and DSS provides an optimal extension of human and animal biomonitoring due to the non-invasive sample collection and easy sample preparation.

## Electronic supplementary material

Below is the link to the electronic supplementary material.

ESM 1 (PDF 1195 kb)

## Abbreviations

2’*R*-OTA: 2’*R*-Ochratoxin A
10-OH-OTA: 10-Hydroxyochratoxin A
AFB_1_: Aflatoxin B_1_
AFB_2_: Aflatoxin B_2_
AFG_1_: Aflatoxin G_1_
AFG_2_: Aflatoxin G_2_
AFM_1_: Aflatoxin M_1_
ALT: Altenuene
AME: Alternariol monomethyl ether
AOH: Alternariol
BEA: Beauvericin
DBS: Dried blood spot
DH-CIT: Dihydrocitrinone
DON: Deoxynivalenol
DON-3-GlcA: DON-3-glucuronide
DSS: Dried serum spot
EnA: Enniatin A
EnA_1_: Enniatin A_1_
EnB: Enniatin B
EnB_1_: Enniatin B_1_
FB_1_: Fumonisin B_1_
HT-2: HT-2 toxin
HT-2-4-GlcA: HT-2-toxin-4-glucuronide
OTA: Ochratoxin A
OTα: Ochratoxin α
sMRM: Scheduled multiple reaction monitoring
T-2: T-2 toxin
ZAN: Zearalanone
ZEN: Zearalenone
