# Supplementary material for: Multi-mycotoxin analysis using dried blood spots and dried serum spots
Source: Anal Bioanal Chem. 2017 Mar 15;409(13):3369–82. doi: 10.1007/s00216-017-0279-9 (PMC5395583; doi:10.1007/s00216-017-0279-9)
Supplement: Supplementary file 1 — (PDF 1195 kb) [file 216_2017_279_MOESM1_ESM.pdf]

Analytical and Bioanalytical chemistry

Electronic Supplementary Material

Multi-mycotoxin analysis using dried blood spots and dried serum spots

Bernd Osteresch, Susana Viegas, Benedikt Cramer, Hans-Ulrich Humpf

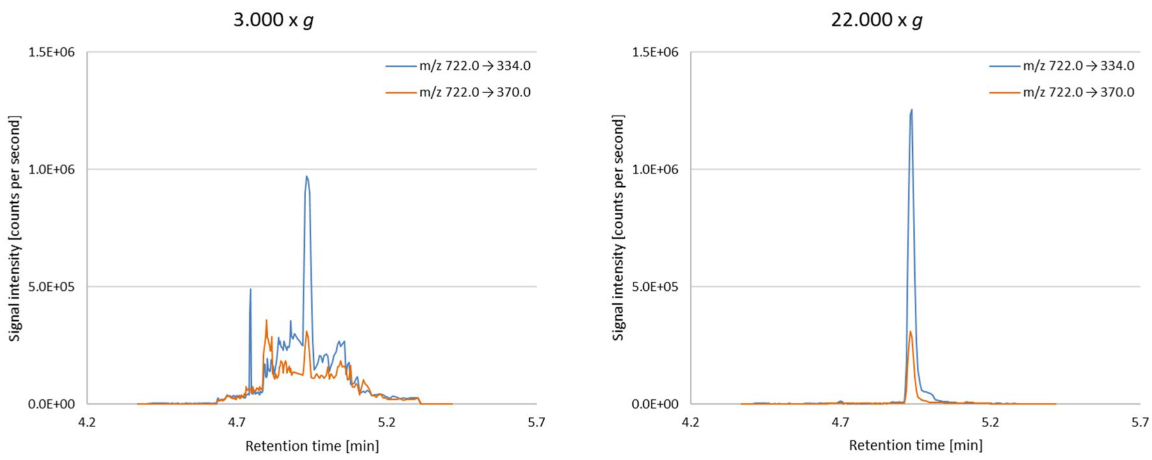

Fig. S1 Comparison of different applied centrifugal forces on the detection of Fumonisin B_1 in extracted whole blood matrix (c_{FB1} = 10 ng/mL)

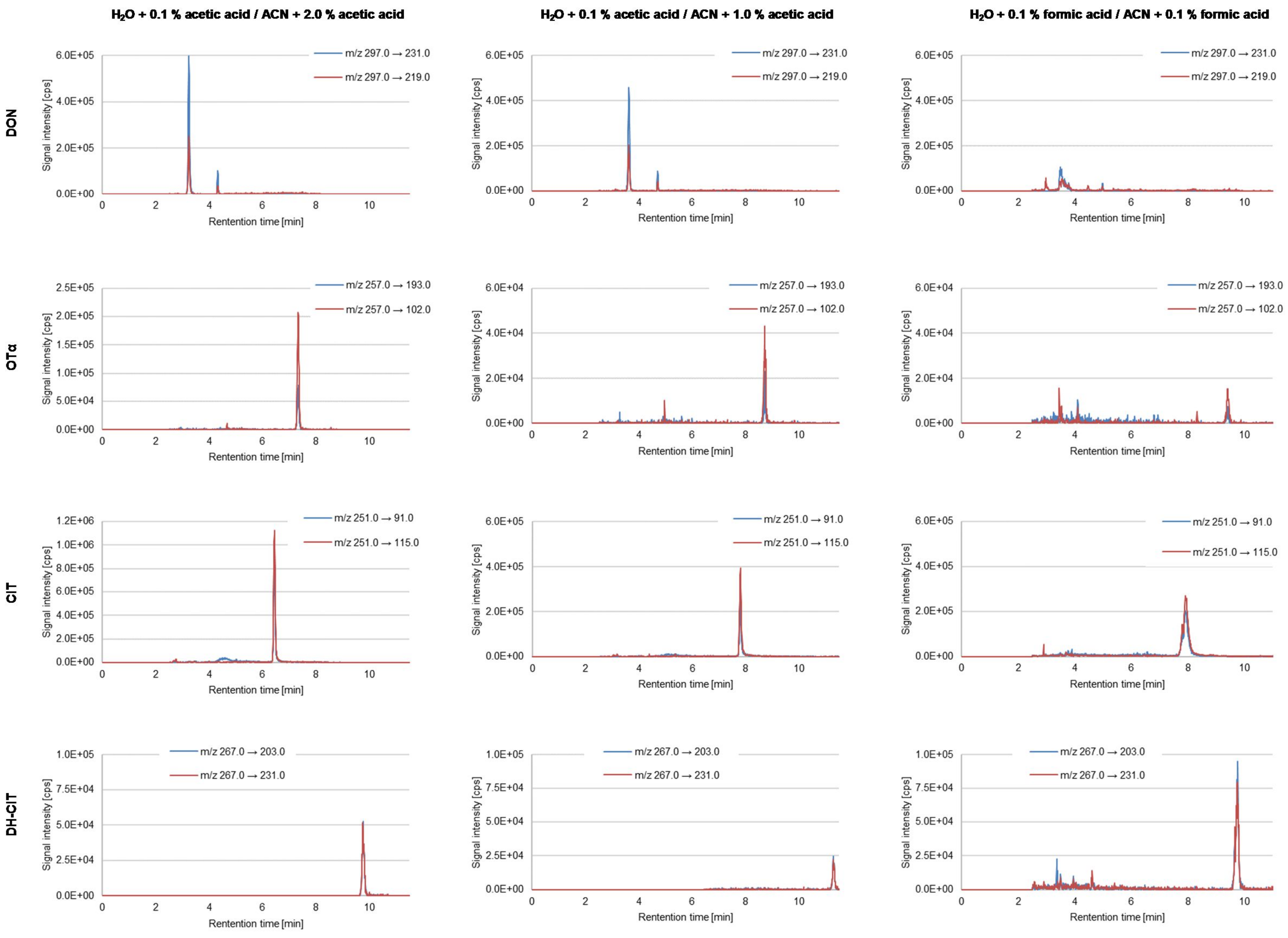

Fig. S2 Influence of eluent additive on the chromatography of DON, OTα, CIT and DH-CIT; column material Gravity SB; for gradient see Experimental section; c_{DON} = 25 ng/mL; $c_{OT\alpha}$ = 25 ng/mL; c_{CIT} = 25 ng/mL; $c_{DH\text{-}CIT}$ = 25 ng/mL

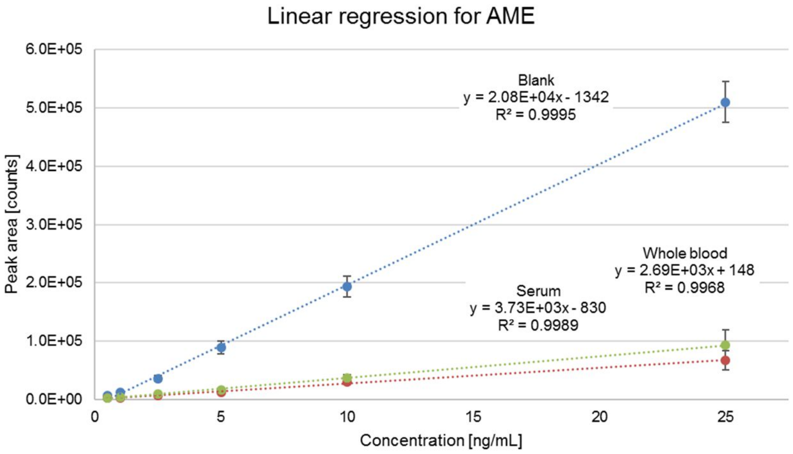

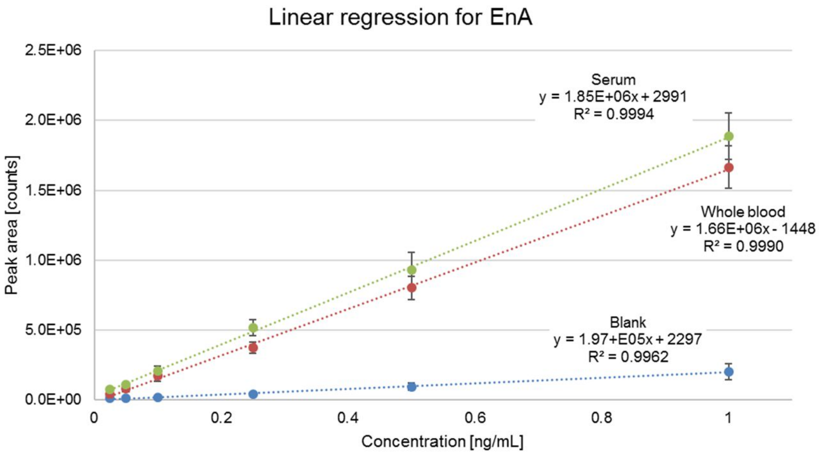

Fig. S3 Calibration curves for AME and EnA; Blue linear regression: neat calibration solution, green: matrix-matched calibration for serum and red: whole blood calibration

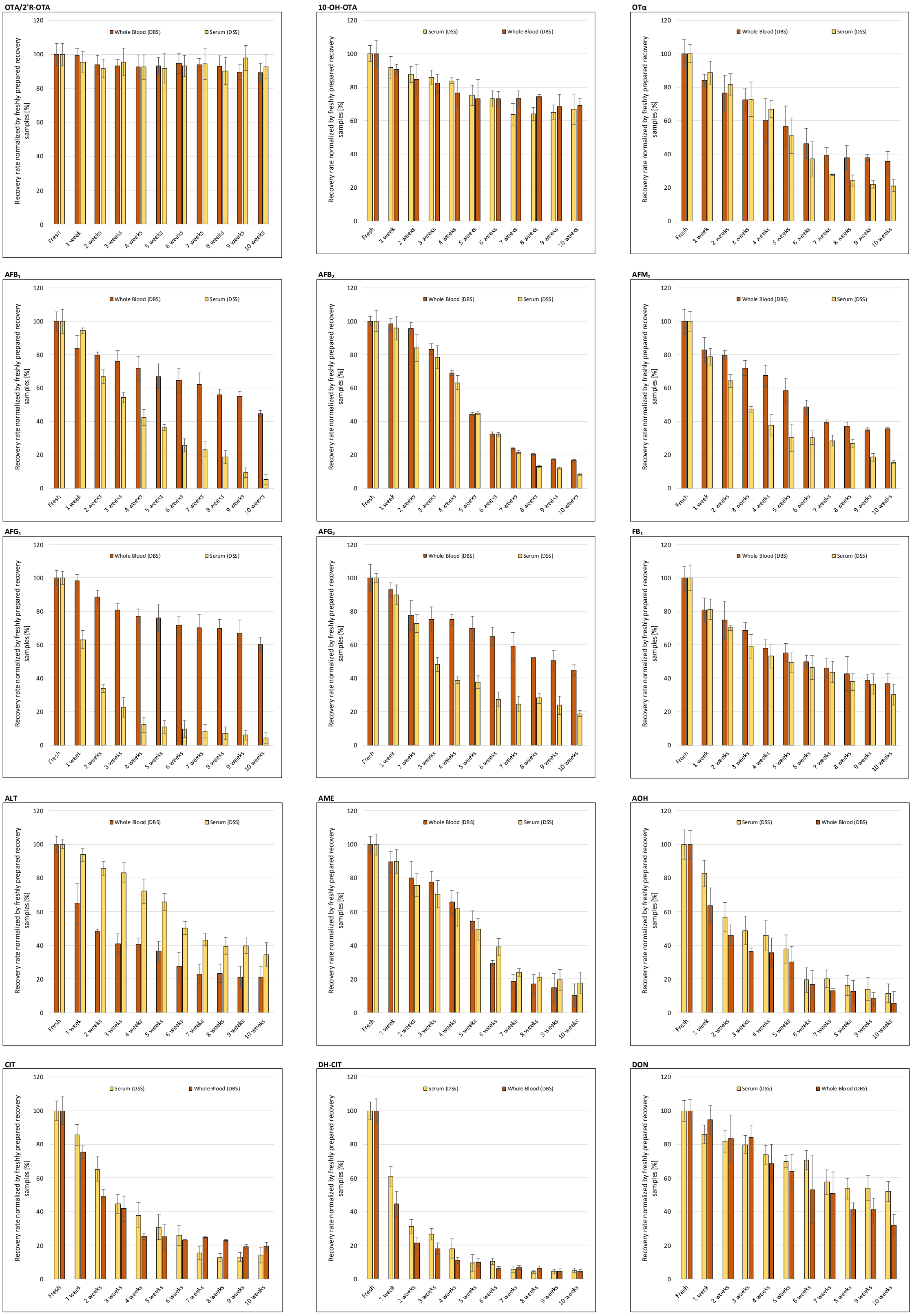

Fig. S4 Relative recovery rates after storage at room temperature (20 °C) in the dark for OTA/2'*R*-OTA, 10-OH-OTA, OTα, AFB_1, AFB_2, AFG_1, AFG_2, AFM_1, FB_1, ALT, AME, AOH, CIT, DH-CIT and DON

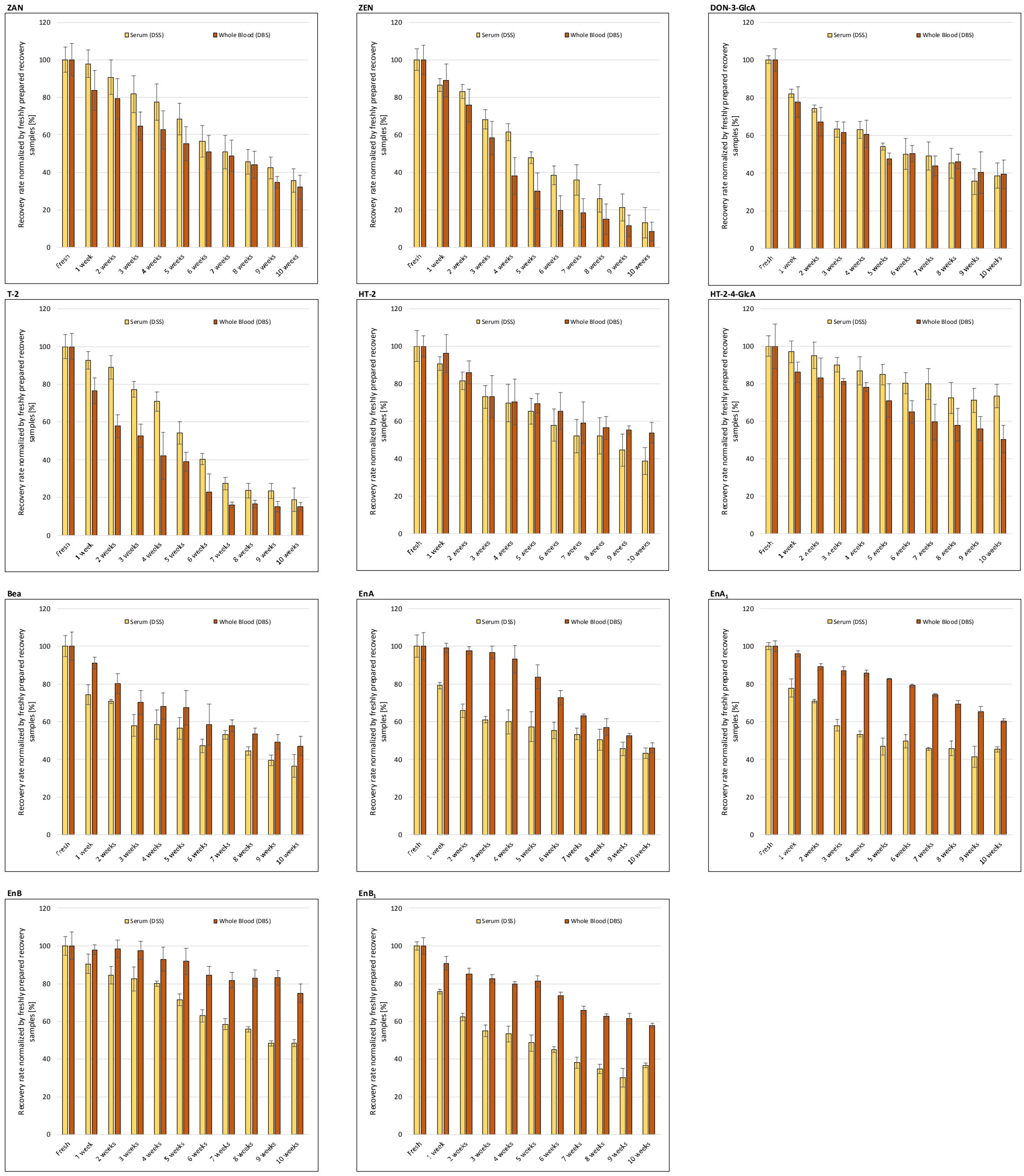

Fig. S5 Relative recovery rates after storage at room temperature (20 °C) in the dark for ZAN, ZEN, DON-3-GlcA, T-2, HT-2, HT-2-GlcA, Bea, EnA, EnA_1, EnB and EnB_1

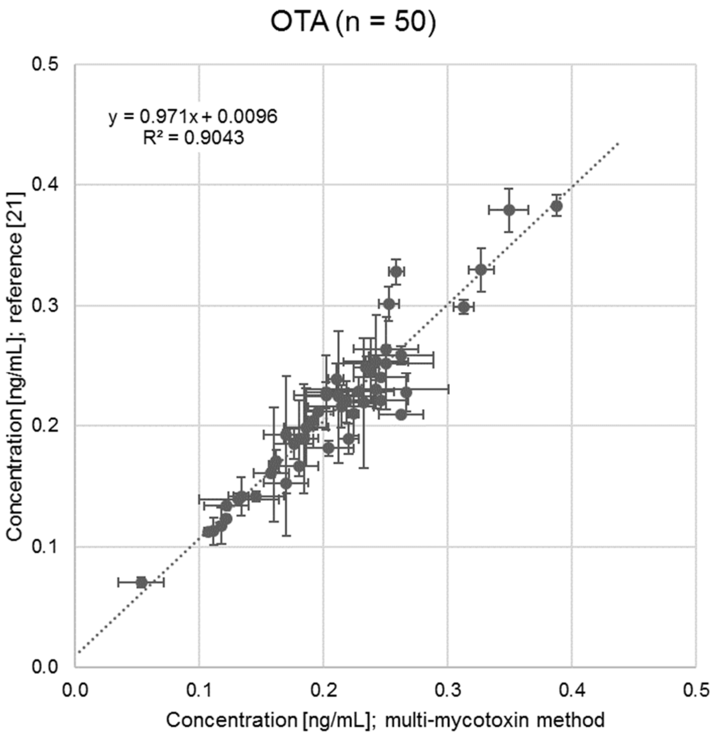

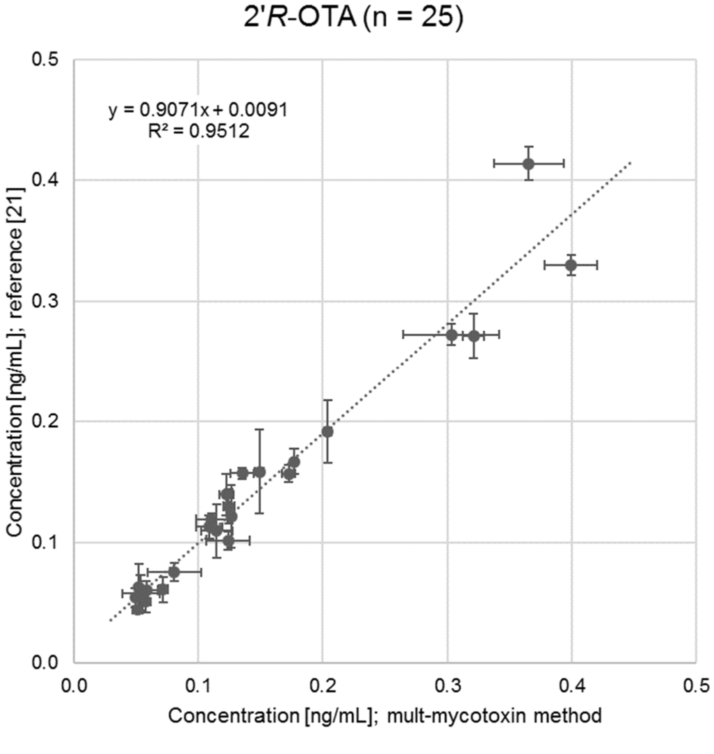

Fig. S6 Correlation of positive findings for OTA and 2’*R*-OTA in DBS (n = 50) between the results from reference [21] (y-axis) and the data obtained by application of the multi-mycotoxin method (x-axis) reported here

Table S1 Comparison of five different blood samples (I-V) spiked with the highest level in triplicate with the prior study results. Calibration was carried out by use of a further matrix sample for matrix-matched calibration. Recovery rate ± RSD is given in [%]

	Recovery rate ± RSD [%]<br>Sample ID					Average of I-V	*Table 3: Average* at highest level	Fortified level [ng/mL]
	I	II	III	IV	V			[ng/mL]
AFB1	108 ± 3.4	103 ± 3.7	111 ± 9.6	125 ± 6.0	105 ± 9.3	**111 ± 7.9**	116±2.8	1
AFB2	115 ± 5.2	110 ± 9.5	119 ± 5.1	126 ± 4.7	115 ± 5.1	**117 ± 4.9**	113±4.5	1.2
AFG1	85 ± 3.7	82 ± 5.5	76 ± 3.1	90 ± 4.4	98 ± 4.0	**86 ± 9.9**	82±7.4	4.5
AFG2	96 ± 2.5	84 ± 9.2	81 ± 3.9	105 ± 9.8	105 ± 7.9	**94 ± 11.8**	97±1.2	1.2
AFM1	98 ± 5.9	104 ± 4.7	120 ± 4.7	103 ± 5.7	116 ± 8.0	**108 ± 8.6**	114±0.9	4.5
ALT	92 ± 4.1	110 ± 10.0	115 ± 4.8	123 ± 6.4	108 ± 12.1	**109 ± 10.3**	106±2.6	10
AME	91 ± 8.3	98 ± 11.2	118 ± 10.7	107 ± 11.4	102 ± 14.8	**103 ± 9.7**	99±2.3	10
AOH	89 ± 9.5	85 ± 5.2	84 ± 10.9	77 ± 6.7	85 ± 8.6	**84 ± 5.1**	88±2.4	10
Bea	88 ± 9.4	82 ± 7.4	93 ± 13.5	89 ± 9.8	88 ± 8.9	**88 ± 4.5**	81±3.7	1
CIT	78 ± 5.0	86 ± 6.6	92 ± 4.3	85 ± 4.0	87 ± 4.9	**86 ± 5.8**	81±2.2	5
DH-CIT	96 ± 2.4	92 ± 3.1	105 ± 4.6	114 ± 7.3	99 ± 5.5	**101 ± 8.5**	94±0.8	25
DON	80 ± 4.9	85 ± 11.0	85 ± 5.3	83 ± 2.7	88 ± 6.0	**84 ± 3.6**	80±6.7	25
DON-3-GlcA	258 ± 10.4	291 ± 9.9	258 ± 11.9	292 ± 14.2	293 ± 12.2	**279 ± 6.7**	311±13.8	25
EnA	105 ± 4.9	99 ± 10.4	102 ± 5.4	114 ± 9.4	109 ± 5.7	**106 ± 5.7**	102±9.5	0.5
EnA1	81 ± 8.4	83 ± 5.2	81 ± 4.3	83 ± 8.6	88 ± 5.5	**83 ± 3.4**	82±6.0	0.5
EnB	70 ± 7.9	74 ± 2.0	85 ± 5.1	89 ± 2.7	91 ± 3.4	**82 ± 11.2**	89±7.0	0.5
EnB1	73 ± 8.4	74 ± 1.9	94 ± 4.0	93 ± 8.6	92 ± 7.5	**85 ± 12.5**	82±2.4	0.5
FB1	104 ± 2.2	110 ± 4.7	109 ± 2.1	114 ± 3.8	119 ± 4.2	**111 ± 5.2**	106±2.3	25
10-OH-OTA	106 ± 2.9	99 ± 4.0	122 ± 4.0	123 ± 8.0	127 ± 9.0	**115 ± 10.6**	117±1.5	1
HT-2	88 ± 6.1	85 ± 8.5	77 ± 5.3	74 ± 5.5	77 ± 8.6	**80 ± 7.1**	84±2.8	25
HT-2-4-GlcA	124 ± 4.5	113 ± 7.7	133 ± 3.2	133 ± 4.3	132 ± 8.8	**127 ± 7.0**	133±6.4	25
OTA/2'*R*-OTA	95 ± 3.9	92 ± 5.1	114 ± 1.5	113 ± 4.1	112 ± 4.7	**105 ± 10.4**	108±5.4	1
OTα	82 ± 4.8	76 ± 7.1	104 ± 4.9	102 ± 3.8	100 ± 7.9	**93 ± 13.8**	101±1.2	1
T-2	105 ± 4.2	102 ± 2.4	111 ± 7.1	113 ± 4.7	108 ± 5.7	**108 ± 4.2**	128±7.1	25
ZAN	114 ± 13.8	96 ± 12.4	117 ± 4.5	124 ± 3.5	118 ± 9.9	**114 ± 9.3**	113±4.7	25
ZEN	108 ± 5.1	101 ± 10.1	129 ± 8.5	127 ± 10.2	110 ± 5.3	**115 ± 10.6**	114±8.9	25

Table S2 Average relative analyte concentration ± RSD [%] of mycotoxins when stored as dried serum spots for 1, 5, and 10 weeks at room temperature and 24 weeks at 4 °C and -18 °C in the dark. Freshly prepared recovery solutions were set at 100 % and corresponding recovery rate after storage are depicted. Color gradient emphasizes the level of nearly no degradation (≥ 95 %, green) towards the highest (≤ 50 %, red) with color steps of 5 %

Serum (DSS)

Temperature	20 °C			4 °C	-18 °C
Storage time	1 week	5 weeks	10 weeks	24 weeks	24 weeks
AFB1	94.1 ± 1.7	36.3 ± 1.9	5.3 ± 2.9	76.8 ± 5.1	99.6 ± 9.6
AFB2	95.8 ± 7.3	44.9 ± 1.1	8.2 ± 1.4	79.3 ± 11.8	89.5 ± 5.7
AFG1	63.0 ± 5.5	10.6 ± 3.9	4.2 ± 3.0	82.1 ± 5.3	91.8 ± 7.2
AFG2	89.8 ± 6.0	37.5 ± 3.7	18.7 ± 1.9	85.7 ± 5.4	100.7 ± 11.1
AFM1	78.7 ± 5.1	30.2 ± 8.1	15.6 ± 1.7	83.4 ± 13.2	88.3 ± 5.5
ALT	93.9 ± 3.8	65.7 ± 4.9	34.4 ± 7.0	81.7 ± 10.9	95.5 ± 9.1
AME	90.0 ± 7.1	49.6 ± 6.3	17.6 ± 6.5	82.3 ± 10.5	93.0 ± 11.3
AOH	82.6 ± 7.8	37.9 ± 8.4	11.6 ± 5.3	89.8 ± 7.9	99.9 ± 5.4
Bea	74.2 ± 5.1	56.4 ± 5.8	36.5 ± 6.1	81.4 ± 8.7	94.4 ± 9.3
CIT	85.5 ± 6.2	30.8 ± 7.4	14.1 ± 4.7	82.0 ± 7.8	93.1 ± 9.7
DH-CIT	61.1 ± 5.9	9.5 ± 5.0	5.0 ± 1.4	83.6 ± 8.7	90.3 ± 9.5
DON	85.8 ± 5.5	69.9 ± 3.6	51.9 ± 6.0	78.7 ± 4.0	93.8 ± 5.2
DON-3-GlcA	82.2 ± 2.2	54.1 ± 2.0	38.5 ± 6.7	85.4 ± 17.4	93.3 ± 16.3
EnA	79.1 ± 1.8	57.4 ± 8.0	43.3 ± 2.8	80.8 ± 13.5	92.3 ± 9.1
EnA1	77.8 ± 7.4	46.9 ± 4.4	45.2 ± 1.4	86.1 ± 9.5	92.7 ± 7.3
EnB	90.5 ± 5.0	71.5 ± 3.1	48.3 ± 1.9	85.1 ± 8.4	98.6 ± 7.6
EnB1	75.8 ± 1.2	48.5 ± 4.4	36.6 ± 1.2	90.6 ± 5.9	94.9 ± 8.8
FB1	81.2 ± 6.1	49.4 ± 5.7	30.2 ± 6.2	91.9 ± 4.5	96.8 ± 3.8
10-OH-OTA	91.7 ± 6.7	75.1 ± 6.1	66.8 ± 9.0	81.4 ± 6.6	95.8 ± 5.9
HT-2	90.6 ± 3.5	65.3 ± 7.0	38.8 ± 7.2	87.4 ± 13.8	95.3 ± 9.3
HT-2-4-GlcA	97.1 ± 5.8	84.8 ± 5.5	73.4 ± 6.3	85.9 ± 8.2	95.8 ± 7.5
OTA/2'R-OTA	95.5 ± 6.1	91.8 ± 8.6	92.7 ± 7.1	81.6 ± 5.7	97.0 ± 4.7
OTα	88.5 ± 6.9	50.7 ± 10.6	20.9 ± 3.4	77.3 ± 3.7	93.1 ± 6.9
T-2	92.7 ± 4.7	54.1 ± 5.9	18.9 ± 6.3	78.8 ± 5.2	91.4 ± 5.6
ZAN	97.8 ± 7.3	68.2 ± 8.4	35.6 ± 6.3	92.4 ± 9.1	94.8 ± 8.1
ZEN	86.4 ± 3.4	47.7 ± 3.3	13.0 ± 8.2	82.5 ± 5.9	94.5 ± 7.5
